# Biochemical Mechanism of Thai Fermented Soybean Extract on UVB-Induced Skin Keratinocyte Damage and Inflammation

**DOI:** 10.3390/ijms26073418

**Published:** 2025-04-05

**Authors:** Supapit Wongkarn, Teera Chewonarin, Jetsada Ruangsuriya, Sirinya Taya, Pornngarm Dejkriengkraikul, Supachai Yodkeeree

**Affiliations:** 1 Department of Biochemistry, Faculty of Medicine, Chiang Mai University, Chiang Mai 50200, Thailand; supapit_wo@cmu.ac.th (S.W.); teera.c@cmu.ac.th (T.C.); jetsada.ruang@cmu.ac.th (J.R.); pornngarm.d@cmu.ac.th (P.D.); 2 Functional Food Research Unit, Multidisciplinary Research Institute, Chiang Mai University, Chiang Mai 50200, Thailand; sirinya.t@cmu.ac.th; 3 Anticarcinogenesis and Apoptosis Research Cluster, Faculty of Medicine, Chiang Mai University, Chiang Mai 50200, Thailand

**Keywords:** fermented soybean, UVB, HaCaT keratinocyte, skin damage, anti-apoptosis

## Abstract

Ultraviolet B (UVB) radiation is a key factor contributing to photodamage in epidermal cells. This study investigated the protective effects of Thua Nao, a Thai fermented soybean product, against UVB-induced damage in human epidermal keratinocytes (HaCaT) and the underlying mechanisms. Thua Nao extract fractions were prepared using a solvent partition method. We found that the dichloromethane fraction (TN-DC), along with its isoflavones daidzein and glycitein, significantly protected against UVB-induced HaCaT cell death. This protection involved inhibiting caspase-9 and caspase-3 activation, thus preventing apoptosis. Additionally, treatment with TN-DC, daidzein, and glycitein suppressed the UVB-induced production of inflammatory mediators, including interleukin-6 (IL-6), IL-8, inducible nitric oxide synthase, and cyclooxygenase-2. These protective effects were associated with reduced intracellular reactive oxygen species and enhanced the levels of antioxidant enzymes, including superoxide dismutase and glutathione peroxidase 4. Signaling pathway analysis revealed that TN-DC activated the pro-survival ERK1/2 and Akt pathways while decreased the phosphorylation of JNK in UVB-exposed cells. On the other hand, daidzein and glycitein enhanced ERK1/2 activation and reduced the phosphorylation of JNK and p38 MAPKs. The involvement of ERK1/2 and Akt activation in cell survival was confirmed using specific inhibitors. Thus, TN-DC and its isoflavones protects keratinocytes from UVB-induced oxidative damage and inflammation by modulating MAPKs and Akt signaling.

## 1. Introduction

Skin aging is influenced by both intrinsic and extrinsic factors, with ultraviolet A (UVA) and ultraviolet B (UVB) radiation playing a significant role in extrinsic aging, also known as photoaging, which accounts for 80% of skin aging [1]. UVB is particularly damaging to the epidermis and upper dermis, leading to sunburn, premature aging, and skin cancer. This damage is caused by reactive oxygen species (ROS) such as hydrogen peroxide, superoxide anions, and hydroxyl radicals [2]. The accumulation of ROS induces oxidative stress, which damages cellular macromolecules and DNA, leading to gene mutations and further skin damage [3].

The skin utilizes antioxidant enzymes such as superoxide dismutase (SOD), catalase, glutathione peroxidase 4 (GPx4), and heme oxygenase-1 (HO-1) to protect against the destructive effects of ROS on the skin. The imbalance of intracellular redox in the skin during UVB-mediated oxidative stress leading to cellular damage, apoptosis, and inflammation, all of which contribute to photoaging [4]. Apoptosis triggered by UVB occurs via both the intrinsic mitochondrial and extrinsic pathways, involving regulators such as Bcl-2, Bcl-xL, Bax, caspase-9, and caspase-3 [5]. Chronic UVB exposure also increases the production of pro-inflammatory mediators like tumor necrosis factor-α (TNF-α), interleukin-1α (IL-1α), IL-1β, IL-6, IL-8, prostaglandin E2, and nitric oxide, further amplifying inflammation [6].

Oxidative stress induced by UVB radiation activates various signaling pathways, including the mitogen-activated protein kinase (MAPK) and PI3K/Akt pathways, which lead to the production of pro-inflammatory cytokines, matrix metalloproteinases (MMPs), and apoptotic mediators [7,8]. In skin cells, UVB exposure specifically triggers the MAPK pathways, involving key kinases such as extracellular signal-regulated kinase1/2 (ERK1/2), c-Jun N-terminal kinase (JNK), and p38. These pathways regulate cellular processes such as survival, apoptosis, and inflammation. UVB-induced ROS have been shown to activate p38 and JNK, which promote apoptosis through the intrinsic pathway, while also inducing pro-inflammatory mediators [9,10]. On the other hand, the activation of Akt and ERK1/2 promotes cell survival, with ERK1/2 additionally enhancing the expression of antioxidant enzymes [11,12]. Thus, finding agents that regulate the activation of MAPK and Akt pathways presents a potential strategy for protecting against UVB-induced skin damage and inflammation.

There is growing interest in natural compounds that provide protection against UVB-induced skin damage [13]. Thua Nao, a traditional Thai fermented soybean product, is commonly used as a seasoning in northern Thai cuisine. The fermentation of boiled soybeans in Thua Nao, like processes used in Japanese natto and Korean cheonggukjang, which enhance the levels of bioactive aglycone isoflavones, including genistein, daidzein, and glycitein [14]. These isoflavones have demonstrated antioxidant, anti-inflammatory, and anticancer properties [15]. The concentration of these compounds in fermented soybeans is highly dependent on the fermentation process and the microorganisms involved. Notably, *Bacillus* and *Lactobacillus* species are predominant in Thua Nao fermentation [16]. The type and concentration of isoflavones significantly influence the biological activity of the fermented soybean. Although the photoprotective effects of genistein and daidzein have been well-established in vitro and vivo [17,18], the molecular mechanisms underlying the protective effects of Thua Nao extract against UVB-induced skin cell damage and inflammation remain unclear. This study aims to explore the effects of Thua Nao and its isoflavone aglycones on UVB-induced skin cell death and inflammation, focusing on the modulation of key signaling pathways such as MAPKs and PI3K/Akt to elucidate the mechanisms through which Thua Nao may help prevent UVB-induced photoaging and cell death.

## 2. Results

### 2.1. Thua Nao Dichloromethane Fraction (TN-DC) Alleviates UVB-Induced HaCaT Cell Death

**Figure 1:**
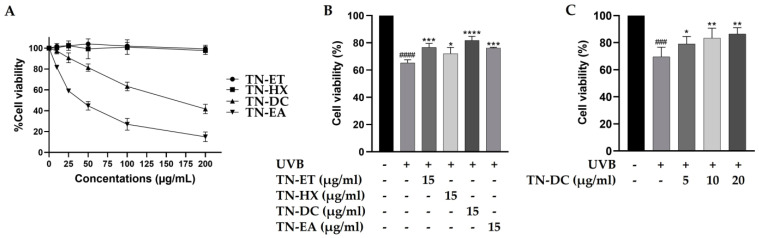
Effects of Thua Nao extract fractions on cell viability in UVB-irradiated HaCaT cells. HaCaT cells were treated with various concentrations of Thua Nao extract fractions for 48 h and the cell viability was measured by SRB assay (**A**). The protective effects of Thua Nao extract fractions on UVB-induced HaCaT cell death. The cells were pretreated with the fractions for 6 h and then irradiated with UVB at 15 mJ/cm^2^. After incubation for 24 h, cell viability was measured using the SRB assay (**B**). The protective effects of different concentrations of TN-DC on UVB-induced damage in HaCaT cells (**C**). * *p* < 0.05, ** *p* < 0.01, *** *p* < 0.005, and **** *p* < 0.001, as compared to the UVB-irradiated alone group; ### *p* < 0.005 and #### *p* < 0.001 as compared to the non-UV group. The experiments were repeated with three biological replicates (*n* = 3) for each condition. The data represents three independent experiments and are presented as mean ± SD.

The cytotoxicity of Thua Nao fractions on HaCaT cells were tested by using the SRB assay. As shown in Figure 1A, the treatment of the cells with the Thua Nao ethanolic extract fraction (TN-ET) and the Thua Nao hexane extract fraction (TN-HX) at 200 μg/mL for 48 h had no significant effect on cell viability. In contrast, the Thua Nao dichloromethane extract fraction (TN-DC) and the Thua Nao ethyl acetate extract fraction (TN-EA) reduced cell viability to 50% at concentrations of 178 μg/mL and 40 μg/mL, respectively. Moreover, the treatment of HaCaT cells with TN-EA at concentrations below 15 μg/mL for 48 h reduced cell viability by less than 20%. Therefore, Thua Nao extract fractions at 15 μg/mL were selected to evaluate their cytoprotective effects against UVB-induced damage in HaCaT cells. In our previous study, UVB irradiation at 15 mJ/cm^2^ induced 42.9% HaCaT cell death. In the current study, UVB irradiation at 15 mJ/cm^2^ reduced HaCaT cell viability to 65.3%, a result consistent with our previous findings [19]. However, the pretreatment of the cells with TN-DC showed the strongest effect by increasing cell viability to 81.8% in UVB-irradiated HaCaT cells (Figure 1B). Furthermore, pretreatment with different concentrations of TN-DC (0–20 µg/mL) significantly reversed UVB-induced HaCaT cell death in a dose-dependent manner (Figure 1C). These results indicate that the TN-DC fraction is the most effective at enhancing cell viability under UVB exposure.

### 2.2. Daidzein and Glycitein in TN-DC Protect HaCaT Cells from UVB-Induced Damage

**Figure 2:**
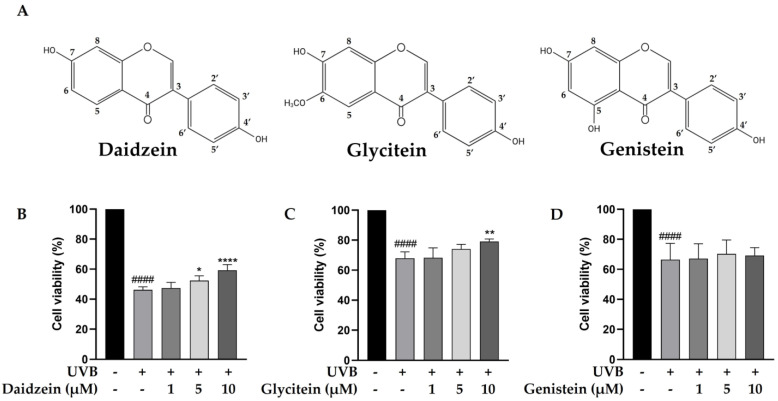
The chemical structures of daidzein, glycitein, and genistein. (**A**) The cytoprotective effects of major isoflavones in TN-DC against UVB-induced damage in HaCaT cells. The cells were pretreated with various concentrations of daidzein (**B**), glycitein (**C**), and genistein (**D**) for 6 h before UVB-irradiation; after 24 h, the cell viability was measured by SRB assay. * *p* < 0.05, ** *p* < 0.01, and **** *p* < 0.001 as compared to the UVB-irradiated alone group. #### *p* < 0.001 as compared to the non-UV group. The experiments were repeated with three biological replicates (*n* = 3) for each condition. The data represent three independent experiments and are presented as mean ± SD.

The phytochemical content in the TN-DC fraction was determined. The total phenolic content in TN-DC was 88.58 ± 1.70 mg GAE/g of extract and the content of total flavonoids was 8.57 ± 0.66 mg CE/g of extract (Table 1). The polyphenol and isoflavone content of TN-DC, including gallic acid, protocatechuic acid, catechin hydrate, 4-hydroxybenzoic acid, caffeic acid, daidzin, ferulic acid, glycitin, genistin, daidzein, glycitein, and genistein was analyzed by using an HPLC assay. Daidzein, glycitein, and genistein were identified as the major constituents of TN-DC, with their retention times consistent with peaks in the chromatogram of the standard compounds including daidzein (33.43 min), glycitein (34.40 min), and genistein (36.32 min) (Figures S1 and S2) and their structures are shown in Figure 2A. The concentrations of daidzein, glycitein, and genistein in TN-DC were 107.85, 26.54, and 150.91 μg/mg of extract, respectively (Table 1). The cytoprotective effects of these compounds against UVB-induced HaCaT cell death were subsequently investigated. As shown in Figure S3, daidzein, glycitein, and genistein, at a concentration of 30 µM, did not affect cell viability. The effective dose of TN-DC (20 μg/mL) contained genistein at a concentration of 11.1 µM. Therefore, isoflavone concentrations below 10 µM were selected to evaluate their cytoprotective effects against UVB-induced damage in HaCaT cells. Treatment with daidzein and glycitein at 10 µM significantly protected HaCaT cells from UVB-induced damage, while genistein showed no such protective effect (Figure 2B–D). These findings indicate that daidzein and glycitein are the active compounds in TN-DC responsible for protecting HaCaT cells from UVB-induced damage.

### 2.3. Protective Effect of TN-DC and Its Active Compounds Against UVB-Induced HaCaT Cell Apoptosis

**Figure 3:**
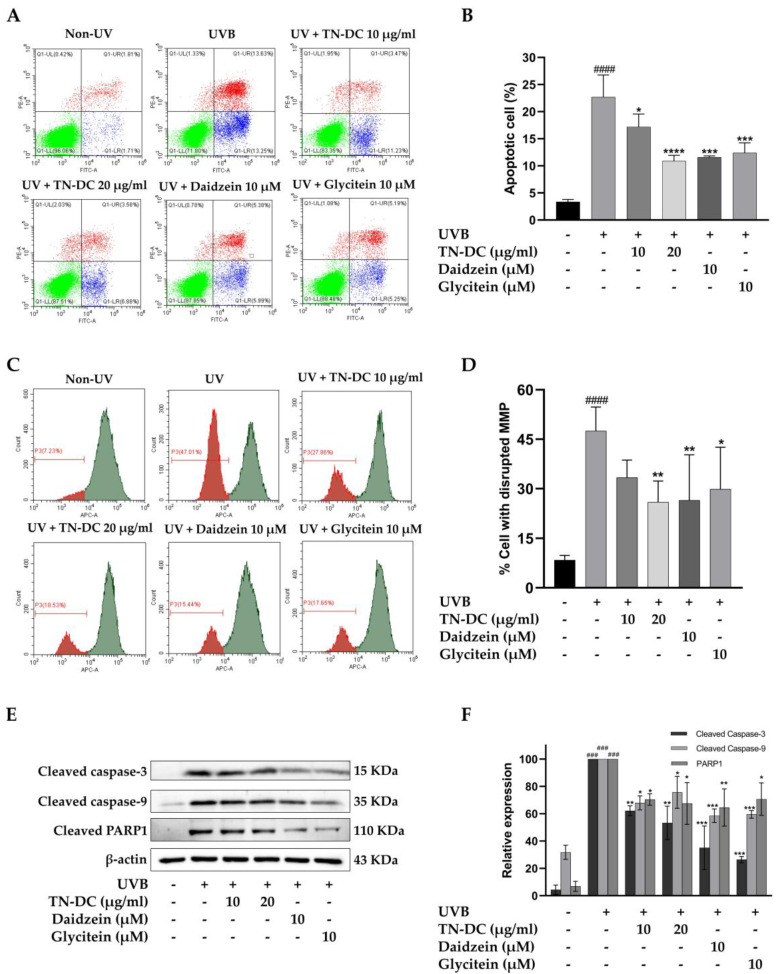
The antiapoptotic effects of TN-DC and its active compounds on UVB-irradiated HaCaT cells. Flow cytometric analysis of apoptosis, including representative histograms (**A**) and quantification (**B**), was performed to evaluate the effects of TN-DC and its active compounds on UVB-irradiated HaCaT cells. The cells were pretreated with TN-DC and its active isoflavones, followed by UVB irradiation at 18 mJ/cm^2^. After 24 h of incubation, apoptosis was assessed using FITC Annexin V/PI flow cytometry. The upper left quadrant represents necrotic cells (PI-positive and Annexin-negative). The upper right quadrant shows late apoptotic cells (Annexin-positive and PI-positive). The lower right quadrant indicates early apoptotic cells (Annexin-positive and PI-negative). The lower left quadrant represents viable cells (Annexin-negative and PI-negative). The effect of TN-DC and its active compounds on MMP. HaCaT cells were pretreated with TN-DC and its isoflavones for 6 h, followed by UVB irradiation. After 18 h of post-irradiation incubation, the mitochondrial membrane potential was assessed using MitoView™ 633 staining. The results are presented as a histogram (**C**) and a bar graph (**D**). The flow cytometry histogram represents the MMP of the cells. The red histogram indicates the population of cells that have lost MMP, whereas the green histogram represents the population of cells with normal MMP. The effects of TN-DC and its bioactive compounds on the UVB-induced expression of apoptotic proteins were analyzed by Western blot at 24 h post-irradiation (**E**). Densitometric and statistical analysis of apoptotic protein expression levels, normalized to β-actin were performed (**F**). All experiments were repeated at least three times. * *p* < 0.05, ** *p* < 0.01, *** *p* < 0.005, and **** *p* < 0.001 compared to the UVB-irradiated group. ### *p* < 0.005 and #### *p* < 0.001 compared to the control group. The experiment of flow cytometry was repeated with two biological replicates (*n* = 2) for each condition. The data represents three independent experiments and is presented as mean ± SD.

We evaluated whether TN-DC and its active compounds attenuate UVB-induced apoptosis in HaCaT cells. The cells were treated with TN-DC (0–20 μg/mL), glycitein (10 μM), or daidzein (10 μM) before being irradiated with UVB, and apoptosis was assessed using annexin V and PI staining. As shown in (Figure 3A,B), UVB irradiation increased apoptosis in HaCaT cells to 22.68%. Pretreatment with TN-DC at 20 μg/mL significantly reduced cell apoptosis to 10.90%. Daidzein and glycitein at 10 μM also significantly reduced UVB-induced apoptosis to 11.60% and 12.38%, respectively. UVB irradiation induces intrinsic apoptosis through alterations in mitochondrial membrane potential (MMP). To evaluate these alterations, the MitoView™ 633 stain was used. This membrane permeable dye fluoresces brightly upon accumulating within mitochondria in a manner dependent on membrane potential. Changes in MMP were analyzed using flow cytometry. As shown in (Figure 3C,D), UVB irradiation caused a significant disruption of MMP in HaCaT cells to 47.50%. In contrast, the pretreatment of the cells with TN-DC at 20 μg/mL significantly decreased the loss of MMP to 25.92%. Furthermore, treatment with daidzein and glycitein at 10 μM significantly reduced MMP disruption to 26.47% and 29.85%, respectively. The involvement of the caspase pathway in UVB-induced apoptosis has been well documented. Therefore, the effects of TN-DC and its active compounds on UVB-induced apoptotic protein expression were investigated. As shown in (Figure 3E,F), UVB irradiation increased the expression of cleaved caspase-3, caspase-9, and PARP1. Notably, pretreatment with TN-DC (20 μg/mL), daidzein (10 μM), and glycitein (10 μM) significantly inhibited UVB-induced cleavage of caspase-3, caspase-9, and PARP1. Collectively, these findings suggest that TN-DC, along with its active compounds daidzein and glycitein, provides a protective effect against UVB-induced apoptosis by regulating the activation of caspase-3, caspase-9 and PARP1.

### 2.4. TN-DC and Its Active Compounds Inhibit UVB-Induced Inflammation in HaCaT Cells

**Figure 4:**
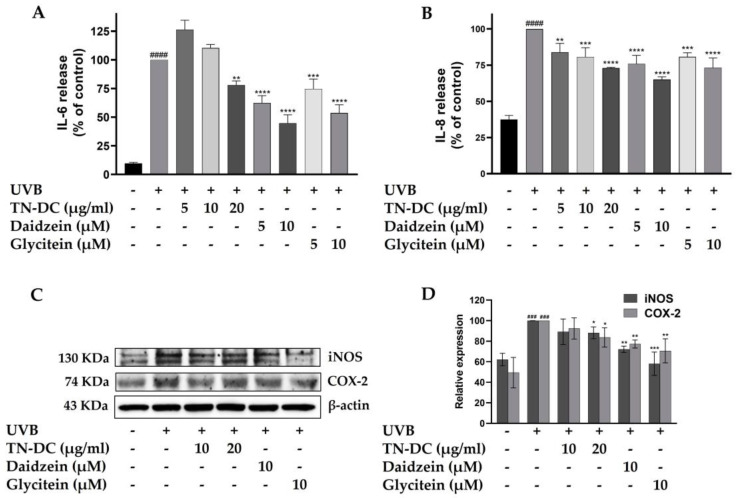
The inflammation-modulating effects of TN-DC and its active compounds on UVB-irradiated HaCaT cells. HaCaT cells were treated with various concentrations of TN-DC and its active compounds for 6 h prior to UVB irradiation. Culture supernatants were collected 24 h post-irradiation, and pro-inflammatory cytokine levels were measured using ELISA. The production of IL-6 (**A**) and IL-8 (**B**) was quantified. Additionally, the effects of TN-DC and its bioactive compounds on the UVB-induced expression of pro-inflammatory enzymes, iNOS, and COX-2 were analyzed by Western blot analysis at 24 h post-irradiation (**C**). Densitometric and statistical analyses of relative protein expression levels were normalized to β-actin (**D**). All experiments were repeated at least three times. * *p* < 0.05, ** *p* < 0.01, *** *p* < 0.005, and **** *p* < 0.001 compared to the UVB-irradiated group. ### *p* < 0.005 and #### *p* < 0.001 compared to the control group. The ELISA experiment was repeated with two biological replicates (*n* = 2) for each condition. The data represents three independent experiments and is presented as mean ± SD.

UVB radiation triggers the production of pro-inflammatory factors within epidermal keratinocytes, which can subsequently disrupt skin barrier function and lead to skin damage. To determine whether TN-DC, daidzein, and glycitein can inhibit UVB-induced pro-inflammatory mediators in HaCaT cells, the expression levels of pro-inflammatory cytokines, including IL-6 and IL-8, as well as pro-inflammatory enzymes such as iNOS and COX-2, were analyzed. Consistent with previous findings, UVB irradiation increased the levels of IL-6 and IL-8. However, TN-DC, daidzein, and glycitein significantly reduced UVB-induced IL-6 and IL-8 levels in a dose-dependent manner (Figure 4A,B). Western blot analysis was performed to assess the expression of iNOS and COX-2. The expression of iNOS and COX-2 proteins was significantly increased following UVB irradiation. However, treatment with TN-DC (20 μg/mL), daidzein (10 μM), and glycitein (10 μM) significantly reduced UVB-induced iNOS and COX-2 expression compared to the UVB-irradiated group (Figure 4C,D). These findings indicated that TN-DC, daidzein, and glycitein exert anti-inflammatory effects by suppressing UVB-induced pro-inflammatory mediator protein expression in HaCaT cells.

### 2.5. Attenuation of UVB-Induced Intracellular ROS in HaCaT Cells by TN-DC and Its Active Compounds

**Figure 5:**
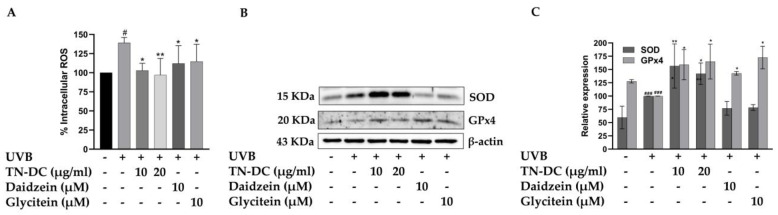
The antioxidant potential of TN-DC and its active compounds on UVB-induced damage in HaCaT cell. The level of intracellular ROS in UVB-irradiated HaCaT cells was investigated by DCF-DA fluorescent dye (**A**). The cells were pretreated with TN-DC and active isoflavones for 6 h and incubated with DCF-DA for 30 min before being UVB irradiated (at 15 mJ/cm^2^). The fluorescence was immediately measured at an excitation/emission of 485/525 nm using a fluorescence microplate reader, after UVB exposure for 1 h. The effect of TN-DC and its bioactive compounds on the expression of antioxidant proteins SOD and GPx4 were evaluated by Western blot (**B**). Densitometric and statistical analyses of protein quantification data, normalized to β-actin, are presented as histograms (**C**). The ABTS and DPPH free radical scavenging activity results are shown in Table 2. All experiments were repeated at least three times. * *p* < 0.05 and ** *p* < 0.01 compared to the UVB-irradiated group. # *p* < 0.05 and ### *p* < 0.005 compared to the control group. The DCF-DA experiment was repeated with three biological replicates (*n* = 3) for each condition. The data represents three independent experiments and are presented as mean ± SD.

UVB-induced overproduction of ROS can activate pro-inflammatory signaling pathways and trigger cell apoptosis. Therefore, intracellular ROS generation was assessed to evaluate the effects of TN-DC, daidzein, and glycitein on UVB-induced oxidative stress in HaCaT cells. Intracellular ROS generation was detected using a DCF-DA fluorescent probe. As shown in Figure 5A, UVB-irradiated cells exhibited a significant increase in intracellular ROS to 139.10% when compared to non-UVB-irradiated cells. However, treatment with TN-DC at 10 and 20 μg/mL significantly reduced UVB-induced intracellular ROS levels to 102.94% and 96.97%, respectively. Furthermore, daidzein and glycitein at 10 μM significantly decreased UVB-induced ROS production in HaCaT cells. Intracellular oxidative stress can be scavenged by endogenous antioxidant enzymes, including SOD and GPx4. Therefore, the effects of TN-DC and its active compounds on the expression of SOD and GPx4 were analyzed using Western blot. Interestingly, treatment with TN-DC at 10 and 20 μg/mL significantly increased the expression of both SOD and GPx4, whereas daidzein and glycitein at 10 μM increased GPx4 expression but had no effect on SOD expression (Figure 5B,C). The free radical scavenging ability of TN-DC and its active compounds was determined using DPPH and ABTS assays. As shown in Table 2, TN-DC, daidzein, and glycitein exhibited slight DPPH scavenging activity, with IC_50_ values of 380.4 μg/mL, 397.1 μM, and 856.7 μM, respectively, whereas vitamin E had an IC_50_ value of 23.9 μg/mL. Similarly, in the ABTS radical scavenging assay, TN-DC, daidzein, and glycitein showed IC_50_ values of 228.6 μg/mL, 175.4 μM, and 215.3 μM, respectively. In contrast, the positive control, Trolox, showed a significantly lower IC_50_ value of 6.1 μg/mL. These findings demonstrate that TN-DC, daidzein, and glycitein effectively decreased UVB-induced intracellular ROS levels, potentially through the upregulation of antioxidant enzymes expression or slight direct radical scavenging activity.

### 2.6. Regulation of MAPK and Akt Signaling Pathways by TN-DC and Its Active Compounds in UVB-Irradiated HaCaT Cells

**Figure 6:**
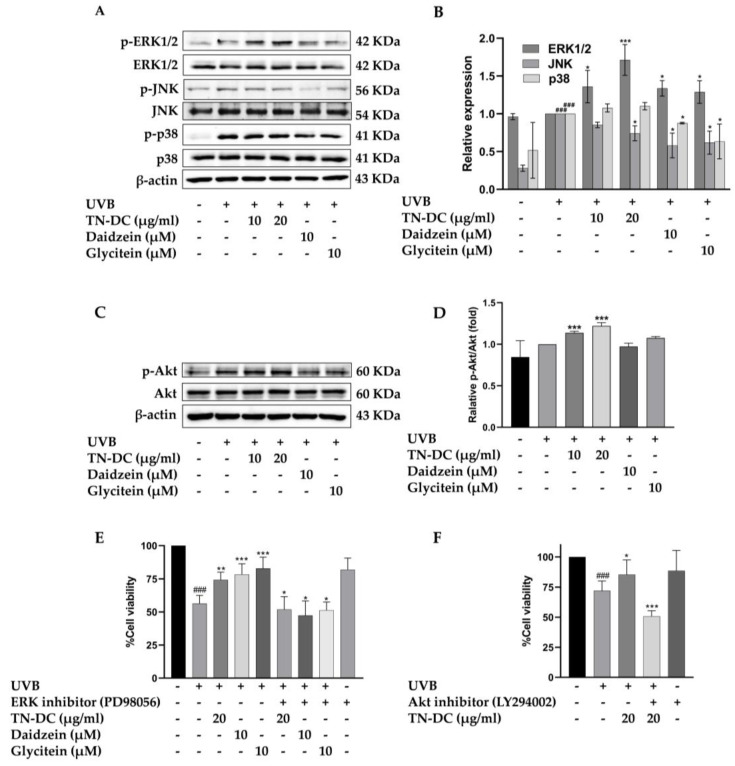
The effect of TN-DC and its active compounds on the modulation of the MAPK and Akt signaling pathways. HaCaT cells were pretreated with TN-DC and its active compounds overnight, followed by UVB irradiation for 1 h. The cells were then collected and subjected to Western blot analysis to assess the activation of MAPKs (**A**) and Akt (**C**). Densitometric and statistical analyses were performed to determine the relative phosphorylation levels of the MAPK (**B**) and Akt (**D**) signaling pathways. To evaluate the role of ERK1/2 and Akt activation in the photoprotective effects of TN-DC and its active compounds against UVB-induced cell damage, the cells were pretreated with TN-DC or its active compounds in the presence of an ERK1/2 inhibitor (PD98056) (**E**) or an Akt inhibitor (LY294002) (**F**), and cell viability was subsequently measured by SRB assay. * *p* < 0.05, ** *p* < 0.01, and *** *p* < 0.005 compared to the UVB-irradiated group treated with the tested compounds. ### *p* < 0.005 compared to the UVB-irradiated alone group. The experiment on cell viability was repeated with three biological replicates (*n* = 3) for each condition. The data represents three independent experiments and are presented as mean ± SD.

To identify the key regulators of TN-DC and its active compounds mediated UVB-induced skin damage, the effect of TN-DC, daidzein, and glycitein on MAPKs and Akt phosphorylation were investigated in HaCaT cells. As shown in (Figure 6A,B), treatment with TN-DC (20 μg/mL), daidzein (10 μM), and glycitein (10 μM) significantly reduced JNK phosphorylation compared to UVB-exposed cells. Meanwhile, daidzein and glycitein decreased p38 phosphorylation, whereas TN-DC had no effect. Interestingly, treatment of the cells with TN-DC, daidzein, and glycitein significantly induced the phosphorylation of ERK1/2 when compared with UVB-irritated cells. To confirm that ERK1/2 activation by TN-DC and its active compounds contributes to their photoprotective effects against UVB-induced cell death, the cells were co-treated with PD98056, an ERK inhibitor, together with TN-DC or its active compounds before UVB irradiation. Cell viability was then assessed. Co-treatment with PD98056 and TN-DC or its active compounds significantly reduced cell viability compared to treatment with TN-DC or its active compounds alone (Figure 6E). In addition, TN-DC significantly increased Akt phosphorylation, whereas daidzein and glycitein had no effect. (Figure 6C,D). By combining the treatment of LY294002 (Akt inhibitor) with TN-DC, cell survival was found to be significantly decreased when compared with TN-DC alone (Figure 6F). Taken together, these results indicate that TN-DC and its active compounds exert photoprotective effects by modulating the MAPK and Akt signaling pathways.

## 3. Discussion

UV radiation is a major environmental factor contributing to skin damage. UVB radiation, which is primarily absorbed by the epidermal layer of the skin, is the main cause of most UV-induced skin damage. Strong evidence suggests UVB irradiation disrupts cell membranes and damages DNA, leading to inflammation and apoptosis [20]. Additionally, oxidative stress from ROS generated by UVB exposure plays a crucial role in these processes [21]. These effects contribute to premature aging, wrinkles, and an increased risk of skin cancer [6]. Therefore, reducing oxidative stress in skin cells is a key strategy for preventing UVB-induced skin damage.

In recent years, there has been growing interest in utilizing natural products to protect human skin from UV-induced damage [22,23]. Soybeans contain various phytochemicals, including isoflavones and phenolic compounds, which exhibit antioxidant properties [24]. Interestingly, fermentation enhances the antioxidant activity of soybean products [25,26]. Fermented soybeans have been shown to reduce UVB-induced inflammation in mice [27]. However, the cytoprotective effects and molecular mechanisms of Thua Nao on UVB-irradiated keratinocytes remain unclear. This study provides the first evidence that the TN-DC fraction protects skin keratinocytes from UVB-induced damage and inflammation.

The antioxidant and photoprotective effects of fermented soybean products are primarily attributed to their isoflavone content. Aglycone isoflavones, particularly genistein and daidzein, are the major compounds found in fermented soybeans [14]. Consistent with our study, TN-DC was enriched with phenolic and flavonoid content, with genistein being the most abundant, followed by daidzein and glycitein. We hypothesize that the high concentration of these key isoflavones in TN-DC contributes to its photoprotective effect on UVB-irradiated keratinocytes. Interestingly, both daidzein and glycitein demonstrated a reduction in UVB-induced HaCaT cell death, with daidzein exhibiting greater potency. This observation is consistent with prior reports documenting that daidzein is a potent agent for reducing UVB-induced skin damage [23,28,29]. While a previous study has reported the protective effects of glycitein against UVB-induced dermal fibroblast cell death [30], our current findings demonstrate, for the first time, its photoprotective effect on UVB-irradiated keratinocytes. Interestingly, genistein did not influence UVB-induced keratinocyte cell death in our experiments. This result contrasts with numerous studies reporting on the photoprotective activity of genistein, which has been observed at high concentrations [28,31,32]. However, the effects of low concentrations remain controversial. For example, a low concentration of genistein showed no inhibitory effect on UVB-induced dermal fibroblast cell death [33]. The biological activity of isoflavones is influenced by the number and position of hydroxyl groups. According to a study by Zielonka et al., polyhydroxy flavones exhibit the following sequence of deprotonation energy: 7-OH > 4′-OH > 5-OH. This indicates that the 5-OH group in genistein is more difficult to deprotonate, thereby reducing its antioxidant activity [34]. Moreover, Iovine et al. demonstrated that daidzein at 10 µM effectively protected against UVB-induced DNA damage, whereas genistein at the same concentration did not exhibit a comparable protective effect [28]. This suggests that the presence of the 5-OH group in genistein may not contribute effectively to UVB-protective activity when compared to daidzein and glycitein. A previous study reported that a combination treatment of HaCaT cells with genistein and daidzein exerted a synergistic photoprotective effect greater than that of each isoflavone alone [28]. In our study, TN-DC also demonstrated greater photoprotective efficacy compared to treatment with the standard alone. In addition, a study by Wang et al. demonstrated the potential use of gallic acid derivatives from *Spirogyra* sp. for the treatment of skin damage caused by UVB [35]. Similarly, a study by Daré et al. investigated the effects of protocatechuic acid in protecting against photodamage and photoaging [36]. These two studies suggest that the synergistic effects of non-isoflavone components in TN-DC may contribute to its photoprotective potential.

Massive apoptosis of skin cells from excessive UVB exposure can disrupt the skin barrier and accelerate photoaging [37,38]. Thus, agents that reduce UVB-induced apoptosis may also contribute to photoprotection. In the present study, we observed that TN-DC, daidzein, and glycitein effectively prevent UVB-induced apoptosis in HaCaT cells. UVB-induced apoptosis involves a complex interplay of DNA damage, oxidative stress, mitochondrial dysfunction, extrinsic death receptor activation, and ER stress [39,40,41,42]. Recent studies demonstrated that UVB-induced cell death mostly occurs through the intrinsic apoptotic pathway, also known as the mitochondrial death signaling pathway [43]. It is well established that UVB-induced oxidative stress can damage mitochondria, leading to alterations in the outer mitochondrial membrane structure and the disruption of the MMP [44]. This permeabilization of the mitochondrial membrane results in the release of cytochrome c, which subsequently triggers downstream events in the apoptotic cascade. Once in the cytosol, cytochrome c interacts with apoptotic protease activating factor-1, forming a complex that recruits and activates procaspase-9 [41]. This initiator caspase plays a crucial role in UV-induced keratinocyte apoptosis by activating the executioner caspase9, caspase-3. In turn, caspase-3 cleaves various cellular proteins, including PARP1 [45]. The activation of PARP1 facilitates cellular disassembly and serves as a hallmark of cells undergoing apoptosis. Consistent with previous reports, our study demonstrates that UVB irradiation induces MMP disruption in HaCaT cells. However, pretreatment with TN-DC, daidzein, and glycitein significantly decreased UVB-induced MMP loss. Additionally, these treatments effectively suppressed the excessive expression of cleaved caspase-9, caspase-3, and PARP1 in UVB-irradiated HaCaT cells. These findings suggest that TN-DC, daidzein, and glycitein may prevent UVB-induced keratinocyte apoptosis by modulating caspase-3 and caspase-9 activation and regulating the mitochondrial apoptotic pathway.

In response to UVB exposure, keratinocytes release various inflammatory mediators, including cytokines and chemokines. A key driver of this inflammatory response is the excessive production of ROS due to UVB-induced oxidative stress, which contributes to skin aging. Elevated levels of pro-inflammatory cytokines such as TNF-α, IL-6, IL-8, and inflammatory enzymes including COX-2 and iNOS are closely associated with the progression of photodamage [46,47]. In accordance with our experimental data, we have confirmed that the expression levels of IL-6, IL-8, COX-2, and iNOS were markedly increased after UVB irradiation in HaCaT cells. Interestingly, treatment of HaCaT cells with TN-DC, daidzein, and glycitein downregulated IL-6 and IL-8 expression in UVB-irradiated cells. This finding is consistent with a study by Lee et al., which demonstrated that dietary fermented soybean and daidzein suppresses UVB-induced inflammation in hairless mice [48]. The dysregulated expression of COX-2 and iNOS has been implicated in UVB-induced skin aging by promoting oxidative stress, chronic inflammation, and ECM degradation [49]. The overproduction of prostaglandin E2, derived from COX-2, and nitric oxide, mediated by iNOS, results in excessive collagen breakdown, sustained inflammation, ultimately accelerating photoaging, wrinkle formation, and skin barrier dysfunction [50]. In this study, TN-DC, daidzein, and glycitein decreased UVB-induced production COX-2 and iNOS. These findings provide evidence indicating that TN-DC attenuates the UVB-induced inflammatory response. Moreover, daidzein and glycitein appear to be the active constituents within TN-DC responsible for suppressing pro-inflammatory mediators.

The generation of free radicals induced by UVB radiation results in the accumulation of excessive ROS, which are cytotoxic to both skin keratinocytes and dermal fibroblasts [51]. These ROS contribute to oxidative stress and cellular damage. This damage can ultimately result in apoptosis and inflammation, playing a crucial role in skin destruction and damage [52]. Consistent with previous reports, our study demonstrated that ROS rapidly accumulated in HaCaT cells following UVB irradiation. However, treatment with TN-DC, daidzein, and glycitein significantly reduced intracellular ROS levels in UVB-exposed cells. This alignment with a study by Chiang et al., which indicates that isoflavone extract from soybean cake inhibited UVB-induced intracellular release of hydrogen peroxide [53]. Skin cells possess various antioxidant defense mechanisms, including low-molecular-weight antioxidants such as glutathione, ascorbic acid, and α-tocopherol, as well as antioxidant enzymes such as SOD, catalase, and GPx4, which counteract ROS-induced oxidative stress [54]. However, excessive exposure to UVB radiation can overwhelm these protective mechanisms, resulting in oxidative damage to cellular constituents leading to skin aging [55]. Interestingly, TN-DC treatment significantly increased the expression levels of SOD-1 and GPx4, while daidzein and glycitein induced GPx4 expression. These effects contribute to an improvement in the redox balance within UVB-irradiated cells. The antioxidant radical scavenging capacity of isoflavones in fermented soybeans has been reported based on DPPH, ABTS, and FRAP assays [56]. Consistent with previous studies, TN-DC, daidzein, and glycitein demonstrate the ability to donate hydrogen atoms to DPPH radicals and transfer electrons to ABTS+ radicals. Interestingly, daidzein and glycitein had low scavenging activity on both DPPH and ABTS+ radicals and glycitein exhibits significantly lower activity compared to daidzein. The weak antioxidant activity of these compounds is also evidenced by recent reports that highlight the importance of the number and position of hydroxyl groups in the isoflavone structure [57]. Melanin plays an important role in protection from the harmful effects of UV radiation. However, excessive melanin production can lead to hyperpigmentation of the skin [58]. Recent studies have reported that daidzein and its metabolite from fermented soy milk can reduce melanin content in α-MSH-stimulated B16 melanoma cells [59]. Similarly, another study found that 7,3,4-THIF and daidzein decreased intracellular melanin content in B16F10 cells [60]. This is an interesting area that we aim to explore in future studies.

Early exposure to UVB radiation results in the generation of ROS, which subsequently activates intracellular signaling pathways, including MAPKs and Akt [61]. These pathways modulate key cellular processes, including cell inflammation, survival, and apoptosis, leading to photoaging and oxidative stress. Upon UVB exposure, JNK and p38 are phosphorylated, initiating downstream processes that promote apoptosis through the intrinsic mitochondrial pathway. This phosphorylation leads to cytochrome c release, which subsequently activates caspase-9 and caspase-3 [62]. Furthermore, UVB-induced JNK and p38 activation also phosphorylates pro-apoptotic Bax and suppresses antiapoptotic Bcl-2 [63]. In addition, several studies have reported that the activation of p38 and JNK can trigger inflammatory mediators in keratinocyte after UVB exposure. Our results demonstrated that UVB induced JNK and p38 activation, and that the treatment of the cells with daidzein and glycitein can attenuate the phosphorylation of JNK and p38. Interestingly, TN-DC treatment specifically inhibited UVB-induced JNK activation but did not affect p38 phosphorylation. These findings suggest the potential of daidzein, glycitein, and TN-DC in modulating the JNK and p38 MAPK signaling pathways to mitigate UVB-induced cellular damage.

On the other hand, the continuous activation of the ERK1/2 and PI3K/Akt signaling pathways has been linked to the apoptotic process. Numerous studies have demonstrated that ERK activation can reduce cell death via induced antiapoptotic proteins [64,65]. In the present study, we observed a significant increase in the levels of phosphorylated ERK1/2 following treatment with TN-DC and its constituent isoflavones. In contrast, only TN-DC, but not daidzein or glycitein, stimulated the phosphorylation of Akt. To confirm the roles of ERK1/2 and PI3K/Akt in the protective effects of TN-DC against UVB-induced cell death, specific inhibitors of ERK1/2 and Akt were used. The results indicate that when HaCaT cells were pretreated with ERK1/2 or Akt inhibitors together with TN-DC, the protective effect of TN-DC against UVB-induced cell death was reversed. Additionally, the combination treatment of daidzein or glycitein with an ERK1/2 inhibitor also reversed their cytoprotective effects against UVB-induced cell death. These findings are consistent with a study by Piao et al., which demonstrated that specific inhibitors of PI3K (LY294002) and ERK1/2 (U0126) reversed the protective effect of rosmarinic acid on UVB-induced HaCaT cell death. Furthermore, the inhibition of the PI3K/Akt and ERK1/2 signaling pathways using chemical inhibitors or the siRNA-mediated silencing of PI3K and ERK1/2 suppressed the rosmarinic acid-induced upregulation of Nrf2 and glutathione synthetase expression [66]. This supports the accumulating evidence indicating that the ERK1/2 and PI3K/Akt signaling pathways play a critical role in the regulation of antioxidant enzyme expression including SOD, HO-1, glutathione synthetase, and GPx4 [67]. Based on what has been mentioned above, we suggest that TN-DC and its active isoflavones induce SOD and GPx4 expression by modulating the ERK1/2 and PI3K/Akt signaling pathways. This regulation is crucial for maintaining cellular homeostasis and adaptation, ultimately promoting HaCaT cell survival and inhibiting UVB-induced cell death.

## 4. Materials and Methods

### 4.1. Chemical and Reagents

Dulbecco’s modified Eagle’s medium (DMEM), penicillin/streptomycin, and trypsin EDTA were purchased from Gibco (Grand Island, NY, USA). Fetal bovine serum (FBS) was supplied by Hyclone (Logan, UT, USA). MitoViewTM 633 was purchased from Biotium (Fremont, CA, USA). The IL-6 and IL-8 ELISA kits and FITC Annexin V kit were obtained from BioLegend (San Diego, CA, USA). LY294002, PD98059, antibodies specific to β-actin, COX-2, iNOS, ERK, p38, p44/42 (ERK), p-p38, and p-p44/42 (ERK) were obtained from Cell Signaling Technology (Danvers, MA, USA). Antibodies specific to active Caspase3, Caspase9, PARP1, p-Akt, Akt, p-JNK, JNK, HO-1, SOD1, and GPX4 were purchased from Abclonal (Woburn, MA, USA). Antibodies specific to iNOS were purchased from Thermo Fisher Scientific (Rockford, IL, USA). The DCF-DA was obtained from Sigma (St. Louis, MO, USA). HPLC standards including genistein, daidzein, glycitein, genistin, daidzin, and glycitin (all with HPLC purity >98%) were obtained from Biopurify Phytochemicals (Chengdu, China). In addition, HPLC standards of gallic acid, protocatechuic acid, catechin hydrate, 4-hydroxybenzoic acid, caffeic acid, and ferulic acid (all with HPLC purity >98%) were obtained from Sigma-Aldrich (St. Louis, MO, USA). Nitrocellulose membrane and ECL reagent were supplied by GE Healthcare (Little Chalfont, UK).

### 4.2. Plant Materials and Sample Preparation

Thua Nao was prepared by the traditional method. Briefly, 1 kg of soybean was soaked in water for 12 h. After soaking, the soybeans were subjected to boiling for a period of 3 h and allowed to cool down to 40 °C. The cooked soybeans were then subjected to a natural fermentation process for 3 days at 37 °C and 80% humidity. Thua Nao was air-dried at 50 °C in a hot air oven and powdered. A ground powder (500 g) was extracted twice with 80% (*v*/*v*) ethanol overnight at room temperature. The solvent was then filtered, concentrated under vacuum evaporation, and freeze-dried to obtain the ethanolic fraction. The ethanolic fraction (TN-ET) was subsequently fractionated using a different polarity of organic solvents including hexane, dichloromethane, and ethyl acetate, and was then concentrated under vacuum evaporation and air-dried to obtain the hexane (TN-HX), dichloromethane (TN-DC), and ethyl acetate (TN-EA) fraction.

### 4.3. Total Phenolic Contents

The modified Folin–Ciocalteu technique were used to determine total phenolic content [68]. TN-DC was dissolved in DMSO then mixed with 10% (*v*/*v*) Folin–Ciocalteu reagent and incubated in the dark for 3 min at room temperature. After that, the mixture was mixed with 300 μL of 7.5% (*w/v*) sodium carbonate (Na_2_CO_3_) and incubated for 30 min. After incubation, the absorbance was measured at 765 nm by spectrophotometry and compared to the gallic acid (GA) standard curve for the determination of total phenolic content.

### 4.4. Total Flavonoid Contents

The aluminum chloride spectrophotometric assay was used to determine the total flavonoid content [69]. TN-DC was dissolved in DMSO and mixed with sodium nitrite (NaNO_2_) at room temperature for 5 min, followed by the addition of the sodium hydroxide (NaOH) after 15 min. After incubation, the absorbance was measured at 510 nm by spectrophotometry and compared to the catechin standard curve for the determination of total flavonoid content.

### 4.5. High-Performance Liquid Chromatography (HPLC) Analysis of TN-DC

TN-DC was diluted in ethanol to a final concentration of 10 mg/mL and filtered using a 0.45 µm syringe filter. A 5 μL sample was injected into a reversed-phase C18 column (Zorbex Eclipse Plus C18, 5 μm, 4.6 × 250 mm, Agilent, Santa Clara, CA, USA). The flow rate was set to 1.0 mL/min. The mobile phase consisted of 0.1% trifluoroacetic acid (solvent A) and methanol (solvent B). A gradient elution was applied as follows: From 0 to 50 min, solvent A decreased from 100% to 0%, while solvent B increased from 0% to 100%. Between 50 and 55 min, solvent A was further reduced to 0% and remained stable for 60 min, while solvent B increased to 100% over the same period. Detection was performed using a UV detector at 254, 260, 280, and 320 nm. The fingerprint of the samples was analyzed and compared with standard compounds commonly found in fermented soybeans, including genistein, daidzein, glycitein, genistin, daidzin, glycitin, gallic acid, protocatechuic acid, catechin hydrate, 4-hydroxybenzoic acid, caffeic acid, and ferulic acid. The content of each isoflavone was quantified by calculating the peak area under the curve and comparing it with the standard calibration curve.

### 4.6. ABTS (2,2′-Azino-Bis-(Ethylbenthiazoline-6-Sulfonic Acid) Assay

The ABTS radical scavenging assay was performed as in the previous study with slight modifications [70]. ABTS·^+^ cation radicals were generated by reacting 7 mM ABTS in water with 2.45 mM potassium persulfate (K_2_S_2_O_8_) in a 1:1 ratio and stored in the dark at room temperature for 12–16 h before use. Afterward, 10 μL of various concentrations of TN-DC or Trolox (as a positive control) were mixed with 990 μL of the diluted ABTS·^+^ solution and incubated in the dark for 6 min. The absorbance was then measured at 734 nm.

### 4.7. DPPH (2,2-Diphenyl-1-Picryldrazal) Assay

The free radical scavenging ability of the extract was evaluated using the DPPH radical scavenging assay, which measures the hydrogen atom donating capacity of the extracts based on the decolorization of a methanol solution of DPPH [71]. The radical scavenging activity of TN-DC was determined using a modified DPPH assay, as described in previous studies [72]. Briefly, the DPPH solution was diluted with methanol to achieve an appropriate absorbance. TN-DC or Vitamin E (as a positive control) were mixed with 180 μL of the diluted DPPH solution to assess their hydrogen atom donating ability. The absorbance was measured at 540 nm using a microplate reader.

### 4.8. Cell and Cell Culture

Human keratinocyte cells (HaCaT cells) obtained from the American Type Culture Collection (ATCC, Manassas, VA, USA) were cultured in Dulbecco’s modified Eagle’s medium (DMEM) supplemented with 10% fetal bovine serum (FBS) and 1% penicillin/streptomycin and maintained at 37 °C in a humidified 5% carbon dioxide (CO_2_) environment. For Thua Nao extract fractions and isoflavones treatment, the compounds were dissolved in DMSO, and then diluted with the culture medium, ensuring that the final concentration of DMSO was less than 0.5% (*v*/*v*).

### 4.9. Cell Viability Assay

Cell viability was measured using the sulforhodamine B (SRB) assay. HaCaT cells were seeded in a 96-well plate at a density of 3 × 10^3^ cells per well and incubated at 37 °C in a humidified atmosphere with 5% CO_2_ overnight. The cells were treated with various concentrations of Thua Nao extract fractions (0–100 µg/mL) or isoflavones (0–50 μM) for 48 h. After incubation, the cells were fixed by adding 100 μL of 10% (*w*/*v*) trichloroacetic acid (TCA) and incubated at 4 °C for 1 h. The plates were then washed slowly with deionized water and dried. The cells were stained with 0.054% (*w*/*v*) sulforhodamine B (SRB) solution for 30 min at room temperature, followed by washing three times with 200 μL of 1% (*v*/*v*) acetic acid to remove excess dye. The protein-bound dye was dissolved in 150 μL of 10 mM Tris-base solution (pH 10.5), and absorbance was measured at 540 nm using a microplate reader.

### 4.10. Sample Treatment and UVB-Irradiation

HaCaT cells were seeded in a 24-well plate at a density of 5 × 10⁴ cells per well and incubated overnight at 37 °C in a humidified atmosphere with 5% CO_2_. The cells were washed with 500 μL of PBS before adding 200 μL of various concentrations of TN-DC in PBS. The cells were then irradiated with UVB at 15 mJ/cm^2^ using a UVB generator (UVP CL-1000 Ultraviolet Crosslinker, Upland, CA, USA) that emits 302 nm UVB radiation. The UV dose was calibrated using a VLX-3W radiometer. After UVB exposure, PBS was removed, and the cells were incubated with 0.5% FBS DMEM containing different concentrations of TN-DC for 24 h. Cell viability was then assessed using the SRB assay.

### 4.11. Apoptosis Evaluation

Apoptotic cell populations were detected using a commercial FITC Annexin V kit (BioLegend, San Diego, CA, USA; Cat. No. 420201). Briefly, the cells were pretreated with TN-DC and its active isoflavones, followed by UVB irradiation at 15 mJ/cm^2^. After 24 h of incubation, the cells were collected, washed with PBS, and stained with 2.5 µL of propidium iodide (PI) and 2.5 µL of Annexin V-FITC at room temperature for 15 min. The stained cells were then analyzed using a flow cytometer, and data were processed with CytExpert for DxFLEX 2.0 software.

### 4.12. Mitochondrial Membrane Potential Quantification

Mitochondrial membrane potential was assessed using MitoViewTM 633 (Biotium, Fremont, CA, USA), according to the manufacturer’s protocol. Briefly, the cells were pretreated with TN-DC and its active isoflavones, followed by UVB irradiation at 15 mJ/cm^2^. After UVB irradiation, the cells were incubated in 0.5% FBS DMEM supplemented with various concentrations of TN-DC and active isoflavones for 18 h. The cells were collected and incubated for 20 min in the dark with 75 nM MitoView™ M 633 in incomplete DMEM at 37 °C in a humidified atmosphere containing 5% CO_2_. After incubation, the cells were washed once with PBS and fluorescence was analyzed using flow cytometry (Beckman Coulter DxFLEX) with 638/660 nm excitation/emission. Data analysis was performed using CytExpert software, version 2.5 for DxFLEX Flow Cytometers.

### 4.13. Enzyme-Linked Immunosorbent Assay (ELISA)

HaCaT cells were pretreated with TN-DC and its active isoflavones, followed by UVB irradiation at 15 mJ/cm^2^. After 24 h of incubation, culture supernatants were collected, and the levels of IL-6 and IL-8 in the culture medium were quantified using ELISA kits (BioLegend, San Diego, CA, USA) according to the manufacturer’s instructions. Absorbance was measured at 450 nm using a microplate reader.

### 4.14. Intracellular ROS Production

HaCaT cells were seeded at a density of 7.5 × 10^4^ cells per well in 24-well plates and incubated overnight. The cells were then pretreated with the indicated concentrations of TN-DC and standard compounds overnight. Subsequently, the treated cells were incubated with 5 μM DCF-DA for 30 min at 37 °C. After incubation, they were washed twice with 500 μL of PBS and exposed to UVB irradiation (15 mJ/cm^2^) in the presence of TN-DC and active compounds in PBS. Fluorescence kinetics were measured every 5 min for up to 60 min at an excitation/emission wavelength of 485/525 nm using a fluorescence microplate reader.

### 4.15. Western Blot Analysis

HaCaT cells were plated in 6-well plates at a density of 2 × 10⁶ cells per well and incubated overnight. The following day, cells were pretreated with the indicated concentrations of TN-DC and standard compounds overnight. Subsequently, 200 μL of various concentrations of TN-DC in PBS were added, and cells were irradiated with UVB at 15 mJ/cm^2^, followed by incubation with TN-DC in 0.5% FBS DMEM for the indicated time. After incubation, the cells were harvested by trypsinization and lysed in RIPA buffer. Before loading, total protein quantification was performed using the Bradford method. An equal amount of protein (approximately 15–23 μg/lane) was loaded onto an SDS-PAGE gel, and proteins were transferred to a membrane via electroblotting. To verify protein transfer efficiency and assess loading consistency, the membrane was stained post-transfer using Ponceau S. The membrane was then blocked with 3% BSA for 1 h before incubation with the primary antibody (diluted in 3% BSA) at 4 °C overnight. Following primary antibody incubation, the membrane was washed five times with TBST (4 min per wash) and then incubated with the secondary antibody (diluted in 3% BSA or skim milk) for 2 h. The membrane was washed three times with TBST (10 min per wash) before imaging using the iBright™ FL1500 Imaging System (Thermo Fisher Scientific, Waltham, MA, USA).

### 4.16. Statistical Analysis

All data from three independent experiments were presented as mean ± SD. Statistical analysis was performed by one-way ANOVA with Dunnett’s test using IBM^®^ SPSS^®^ Statistics V.28.0.1.0 (142). Statistical significance was considered at *p*-values lower than 0.05.

## 5. Conclusions

Our study highlights the protective effects of TN-DC and its active isoflavones, daidzein and glycitein, against UVB-induced keratinocyte apoptosis and inflammation. These effects are mediated by reducing ROS production, enhancing antioxidant defenses, and downregulating pro-inflammatory mediators (IL-6, IL-8, COX-2, and iNOS). Additionally, TN-DC modulates MAPKs and Akt signaling pathways, contributing to its antiphotoaging properties. Further in vivo studies are necessary to validate its efficacy and explore TN-DC potential as a therapeutic agent for skin protection.

## Figures and Tables

**Table 1:** The determination of the total phenolic contents, total flavonoid contents, and phytochemical compounds in TN-DC. The experiments were repeated with three biological replicates (*n* = 3) for each condition. The data represents three independent experiments and are presented as mean ± SD.

Compound	TN-DC
Total phenolic (mg of gallic acid/g of extract)	88.58 ± 1.70
Gallic acid (μg/mL)	0.29 ± 0.10
Protocatechuic acid (μg/mL)	ND
Catechin hydrate (μg/mL)	ND
4-Hydroxybenzoic acid (μg/mL)	ND
Caffeic acid (μg/mL)	ND
Ferulic acid (μg/mL)	ND
Total flavonoid (mg of catechin/g extract)	8.57 ± 0.66
Daidzin (μg/mL)	0.04 ± 0.67
Glycitin (μg/mL)	ND
Genistin (μg/mL)	0.27 ± 0.43
Daidzein (μg/mL)	107.85 ± 10.69
Glycitein (μg/mL)	26.54 ± 2.09
Genistein (μg/mL)	150.91 ± 7.59

ND: Not detected.

**Table 2:** The ABTS and DPPH free radical activity of TN-DC and its active compounds was examined in the present study.

Compound	DPPH Radical Scavenging Activity (IC_50_)	ABTS Radical Scavenging Activity (IC_50_)
Vitamin E (µg/mL)	23.9 ± 8.4	-
Trolox (µg/mL)	-	6.1 ± 1.5
TN-DC (µg/mL)	380.4 ± 46.1	228.6 ± 3.6
Daidzein (µM)	397.1 ± 66.9	175.4 ± 3.4
Glycitein (µM)	856.7 ± 65.6	215.3 ± 23.1

## Data Availability

Data are contained within the article and Supplementary Material.

## Supplementary Materials

The following supporting information can be downloaded at: https://www.mdpi.com/article/10.3390/ijms26073418/s1.

## References

[1] Gromkowska-Kępka K.J., Puścion-Jakubik A., Markiewicz-Żukowska R., Socha K. (2021). The impact of ultraviolet radiation on skin photoaging—Review of in vitro studies. J. Cosmet. Dermatol..

[2] Byun K.-A., Lee S.Y., Oh S., Batsukh S., Jang J.-W., Lee B.-J., Rheu K.-m., Li S., Jeong M.-S., Son K.H. (2024). Fermented Fish Collagen Attenuates Melanogenesis via Decreasing UV-Induced Oxidative Stress. Mar. Drugs.

[3] Lee J.W., Ratnakumar K., Hung K.F., Rokunohe D., Kawasumi M. (2020). Deciphering UV-induced DNA damage responses to prevent and treat skin cancer. Photochem. Photobiol..

[4] Wei M., He X., Liu N., Deng H. (2024). Role of reactive oxygen species in ultraviolet-induced photodamage of the skin. Cell Div..

[5] Zhang J.-A., Luan C., Huang D., Ju M., Chen K., Gu H. (2020). Induction of autophagy by baicalin through the AMPK-mTOR pathway protects human skin fibroblasts from ultraviolet B radiation-induced apoptosis. Drug Des. Dev. Ther..

[6] Tu Y., Quan T. (2016). Oxidative stress and human skin connective tissue aging. Cosmetics.

[7] Hsu W.-H., Chung C.-P., Wang Y.-Y., Kuo Y.-H., Yeh C.-H., Lee I.-J., Lin Y.-L. (2022). Dendrobium nobile protects retinal cells from UV-induced oxidative stress damage via Nrf2/HO-1 and MAPK pathways. J. Ethnopharmacol..

[8] Li Y., Gao J., Liu S., Chen S., Wei X., Guan Y., Li X., Li Y., Huang Z., Li G. (2024). Ergothioneine Protects Against UV-Induced Oxidative Stress Through the PI3K/AKT/Nrf2 Signaling Pathway. Clin. Cosmet. Investig. Dermatol..

[9] Jia R., Cao L.-P., Du J.-L., He Q., Gu Z.-Y., Jeney G., Xu P., Yin G.-J. (2020). Effects of high-fat diet on antioxidative status, apoptosis and inflammation in liver of tilapia (Oreochromis niloticus) via Nrf2, TLRs and JNK pathways. Fish Shellfish Immunol..

[10] Kim S.-H., Yoo E.-S., Woo J.-S., Han S.-H., Lee J.-H., Jung S.-H., Kim H.-J., Jung J.-Y. (2019). Antitumor and apoptotic effects of quercetin on human melanoma cells involving JNK/P38 MAPK signaling activation. Eur. J. Pharmacol..

[11] Balmanno K., Cook S. (2009). Tumour cell survival signalling by the ERK1/2 pathway. Cell Death Differ..

[12] Wang L., Zhang X., Xiong X., Zhu H., Chen R., Zhang S., Chen G., Jian Z. (2022). Nrf2 regulates oxidative stress and its role in cerebral ischemic stroke. Antioxidants.

[13] Zhao C., Wu S., Wang H. (2025). Medicinal Plant Extracts Targeting UV-Induced Skin Damage: Molecular Mechanisms and Therapeutic Potential. Int. J. Mol. Sci..

[14] Yang H.J., Park S., Pak V., Chung K.R., Kwon D.Y. (2011). Fermented soybean products and their bioactive compounds. Soybean Health.

[15] Mun E.-G., Kim B., Kim E.-Y., Lee H.-J., Kim Y., Park Y., Cha Y.-S. (2018). Research trend in traditional fermented foods focused on health functional evaluation. J. Korean Soc. Food Sci. Nutr..

[16] Sivamaruthi B.S., Alagarsamy K., Suganthy N., Thangaleela S., Kesika P., Chaiyasut C. (2022). The role and significance of Bacillus and Lactobacillus species in Thai fermented foods. Fermentation.

[17] Wójciak M., Drozdowski P., Skalska-Kamińska A., Zagórska-Dziok M., Ziemlewska A., Nizioł-Łukaszewska Z., Latalska M. (2024). Protective, Anti-Inflammatory, and Anti-Aging Effects of Soy Isoflavones on Skin Cells: An Overview of In Vitro and In Vivo Studies. Molecules.

[18] Cheon G.-Y. (2024). Photoprotective Effects of Germinated and Fermented Soybean Extract on UVB-Irradiated SKH-1 Hairless Mice. Ph.D. Dissertation.

[19] Charachit N., Sukhamwang A., Dejkriengkraikul P., Yodkeeree S. (2022). Hyperoside and quercitrin in Houttuynia cordata extract attenuate UVB-induced human keratinocyte cell damage and oxidative stress via modulation of MAPKs and Akt signaling pathway. Antioxidants.

[20] Kunchana K., Jarisarapurin W., Chularojmontri L., Wattanapitayakul S.K. (2021). Potential use of amla (Phyllanthus emblica L.) fruit extract to protect skin keratinocytes from inflammation and apoptosis after UVB irradiation. Antioxidants.

[21] Naikoo S., Tasduq S.A. (2020). Trigonelline, a naturally occurring alkaloidal agent protects ultraviolet-B (UV-B) irradiation induced apoptotic cell death in human skin fibroblasts via attenuation of oxidative stress, restoration of cellular calcium homeostasis and prevention of endoplasmic reticulum (ER) stress. J. Photochem. Photobiol. B Biol..

[22] Svobodová A., Psotová J., Walterová D. (2003). Natural phenolics in the prevention of UV-induced skin damage. A review. Biomed. Pap. Med. Fac. Univ. Palacky. Olomouc Czech Repub..

[23] Saewan N., Jimtaisong A. (2015). Natural products as photoprotection. J. Cosmet. Dermatol..

[24] Huang C.-C., Hsu B.-Y., Wu N.-L., Tsui W.-H., Lin T.-J., Su C.-C., Hung C.-F. (2010). Anti-photoaging effects of soy isoflavone extract (aglycone and acetylglucoside form) from soybean cake. Int. J. Mol. Sci..

[25] Adebo O.A., Gabriela Medina-Meza I. (2020). Impact of fermentation on the phenolic compounds and antioxidant activity of whole cereal grains: A mini review. Molecules.

[26] Xiao Y., Wang L., Rui X., Li W., Chen X., Jiang M., Dong M. (2015). Enhancement of the antioxidant capacity of soy whey by fermentation with Lactobacillus plantarum B1–6. J. Funct. Foods.

[27] Lee T.H., Do M.H., Oh Y.L., Cho D.W., Kim S.H., Kim S.Y. (2014). Dietary fermented soybean suppresses UVB-induced skin inflammation in hairless mice via regulation of the MAPK signaling pathway. J. Agric. Food Chem..

[28] Iovine B., Iannella M.L., Gasparri F., Monfrecola G., Bevilacqua M.A. (2011). Synergic effect of genistein and daidzein on UVB-induced DNA damage: An effective photoprotective combination. Biomed Res. Int..

[29] Lin J.Y., Tournas J.A., Burch J.A., Monteiro-Riviere N.A., Zielinski J. (2008). Topical isoflavones provide effective photoprotection to skin. Photodermatol. Photoimmunol. Photomed..

[30] Seo G.Y., Park S., Huh J.-S., Cho M. (2014). The protective effect of glycitin on UV-induced skin photoaging in human primary dermal fibroblast. J. Korean Soc. Appl. Biol. Chem..

[31] Wei H., Saladi R., Lu Y., Wang Y., Palep S.R., Moore J., Phelps R., Shyong E., Lebwohl M.G. (2003). Isoflavone genistein: Photoprotection and clinical implications in dermatology. J. Nutr..

[32] Tang S.-C., Hsiao Y.-P., Ko J.-L. (2022). Genistein protects against ultraviolet B–induced wrinkling and photoinflammation in in vitro and in vivo models. Genes Nutr..

[33] Wang Y.N., Wu W., Chen H.C., Fang H. (2010). Genistein protects against UVB-induced senescence-like characteristics in human dermal fibroblast by p66Shc down-regulation. J. Dermatol. Sci..

[34] Zielonka J., Gębicki J., Grynkiewicz G. (2003). Radical scavenging properties of genistein. Free Radic. Biol. Med..

[35] Wang L., Ryu B., Kim W.-S., Kim G.H., Jeon Y.-J. (2017). Protective effect of gallic acid derivatives from the freshwater green alga Spirogyra sp. against ultraviolet B-induced apoptosis through reactive oxygen species clearance in human keratinocytes and zebrafish. Algae.

[36] Daré R.G., Costa A., Nakamura C.V., Truiti M.C., Ximenes V.F., Lautenschlager S.O., Sarmento B. (2020). Evaluation of lipid nanoparticles for topical delivery of protocatechuic acid and ethyl protocatechuate as a new photoprotection strategy. Int. J. Pharm..

[37] Tang Z., Tong X., Huang J., Liu L., Wang D., Yang S. (2021). Research progress of keratinocyte-programmed cell death in UV-induced skin photodamage. Photodermatol. Photoimmunol. Photomed..

[38] Salminen A., Kaarniranta K., Kauppinen A. (2022). Photoaging: UV radiation-induced inflammation and immunosuppression accelerate the aging process in the skin. Inflamm. Res..

[39] Li L., Ngo H.T., Hwang E., Wei X., Liu Y., Liu J., Yi T.-H. (2019). Conditioned medium from human adipose-derived mesenchymal stem cell culture prevents UVB-induced skin aging in human keratinocytes and dermal fibroblasts. Int. J. Mol. Sci..

[40] Sarkar S., Gaddameedhi S. (2020). Solar ultraviolet-induced DNA damage response: Melanocytes story in transformation to environmental melanomagenesis. Environ. Mol. Mutagen..

[41] He H., Xiong L., Jian L., Li L., Wu Y., Qiao S. (2022). Role of mitochondria on UV-induced skin damage and molecular mechanisms of active chemical compounds targeting mitochondria. J. Photochem. Photobiol. B Biol..

[42] Lee T.-A., Huang Y.-T., Hsiao P.-F., Chiu L.-Y., Chern S.-R., Wu N.-L. (2022). Critical roles of irradiance in the regulation of UVB-induced inflammasome activation and skin inflammation in human skin keratinocytes. J. Photochem. Photobiol. B Biol..

[43] Denning M., Wang Y., Tibudan S., Alkan S., Nickoloff B., Qin J. (2002). Caspase activation and disruption of mitochondrial membrane potential during UV radiation-induced apoptosis of human keratinocytes requires activation of protein kinase C. Cell Death Differ..

[44] Maglio D.H.G., Paz M.L., Ferrari A., Weill F.S., Czerniczyniec A., Leoni J., Bustamante J. (2005). Skin damage and mitochondrial dysfunction after acute ultraviolet B irradiation: Relationship with nitric oxide production. Photodermatol. Photoimmunol. Photomed..

[45] Lee C.-H., Wu S.-B., Hong C.-H., Yu H.-S., Wei Y.-H. (2013). Molecular mechanisms of UV-induced apoptosis and its effects on skin residential cells: The implication in UV-based phototherapy. Int. J. Mol. Sci..

[46] Seo M., Juhnn Y.-S. (2010). Gq protein mediates UVB-induced cyclooxygenase-2 expression by stimulating HB-EGF secretion from HaCaT human keratinocytes. Biochem. Biophys. Res. Commun..

[47] Zattra E., Coleman C., Arad S., Helms E., Levine D., Bord E., Guillaume A., El-Hajahmad M., Zwart E., van Steeg H. (2009). Polypodium leucotomos extract decreases UV-induced Cox-2 expression and inflammation, enhances DNA repair, and decreases mutagenesis in hairless mice. Am. J. Pathol..

[48] Wu P.-Y., Lyu J.-L., Liu Y.-J., Chien T.-Y., Hsu H.-C., Wen K.-C., Chiang H.-M. (2017). Fisetin regulates Nrf2 expression and the inflammation-related signaling pathway to prevent UVB-induced skin damage in hairless mice. Int. J. Mol. Sci..

[49] Fernandes A., Rodrigues P., Pintado M., Tavaria F. (2023). A systematic review of natural products for skin applications: Targeting inflammation, wound healing, and photo-aging. Phytomedicine.

[50] Ansary T.M., Hossain M.R., Kamiya K., Komine M., Ohtsuki M. (2021). Inflammatory molecules associated with ultraviolet radiation-mediated skin aging. Int. J. Mol. Sci..

[51] Chen J., Liu Y., Zhao Z., Qiu J. (2021). Oxidative stress in the skin: Impact and related protection. Int. J. Cosmet. Sci..

[52] Pourzand C., Tyrrell R.M. (1999). INVITED REVIEW-Apoptosis, the role of oxidative stress and the example of solar UV radiation. Photochem. Photobiol..

[53] Chiang H.-S., Wu W.-B., Fang J.-Y., Chen B.-H., Kao T.-H., Chen Y.-T., Huang C.-C., Hung C.-F. (2007). UVB-protective effects of isoflavone extracts from soybean cake in human keratinocytes. Int. J. Mol. Sci..

[54] Rhie G.-e., Shin M.H., Seo J.Y., Choi W.W., Cho K.H., Kim K.H., Park K.C., Eun H.C., Chung J.H. (2001). Aging-and photoaging-dependent changes of enzymic and nonenzymic antioxidants in the epidermis and dermis of human skin in vivo. J. Investig. Dermatol..

[55] Wenk J., Brenneisen P., Meewes C., Wlaschek M., Peters T., Blaudschun R., Ma W., Kuhr L., Schneider L., Scharffetter-Kochanek K. (2001). UV-induced oxidative stress and photoaging. Curr. Probl. Dermatol..

[56] Thaipong K., Boonprakob U., Crosby K., Cisneros-Zevallos L., Byrne D.H. (2006). Comparison of ABTS, DPPH, FRAP, and ORAC assays for estimating antioxidant activity from guava fruit extracts. J. Food Compos. Anal..

[57] Arora A., Nair M.G., Strasburg G.M. (1998). Antioxidant activities of isoflavones and their biological metabolites in a liposomal system. Arch. Biochem. Biophys..

[58] Brenner M., Hearing V.J. (2008). The protective role of melanin against UV damage in human skin. Photochem. Photobiol..

[59] Chang C.-J., Tsai T.-Y. (2016). Antimelanogenic effects of the novel melanogenic inhibitors daidzein and equol, derived from soymilk fermented with Lactobacillus plantarum strain TWK10, in B16F0 mouse melanoma cells. J. Funct. Foods.

[60] Kim J.H., Lee J.-E., Kim T., Yeom M.H., Park J.S., Di Luccio E., Chen H., Dong Z., Lee K.W., Kang N.J. (2020). 7, 3′, 4′-trihydroxyisoflavone, a metabolite of the soy isoflavone daidzein, suppresses α-melanocyte-stimulating hormone-induced melanogenesis by targeting melanocortin 1 receptor. Front. Mol. Biosci..

[61] Prasanth M.I., Gayathri S., Bhaskar J.P., Krishnan V., Balamurugan K. (2020). Understanding the role of p38 and JNK mediated MAPK pathway in response to UV-A induced photoaging in Caenorhabditis elegans. J. Photochem. Photobiol. B Biol..

[62] Dhanasekaran D.N., Reddy E.P. (2017). JNK-signaling: A multiplexing hub in programmed cell death. Genes Cancer.

[63] Mavrogonatou E., Angelopoulou M., Rizou S.V., Pratsinis H., Gorgoulis V.G., Kletsas D. (2022). Activation of the JNKs/ATM-p53 axis is indispensable for the cytoprotection of dermal fibroblasts exposed to UVB radiation. Cell Death Dis..

[64] Ming M., Han W., Maddox J., Soltani K., Shea C.R., Freeman D.M., He Y.-Y. (2010). UVB-induced ERK/AKT-dependent PTEN suppression promotes survival of epidermal keratinocytes. Oncogene.

[65] Li D.-Y., Tao L., Liu H., Christopher T., Lopez B., Ma X. (2006). Role of ERK1/2 in the anti-apoptotic and cardioprotective effects of nitric oxide after myocardial ischemia and reperfusion. Apoptosis.

[66] Kim Y.R., Hyun J.W. (2024). Rosmarinic Acid Inhibits Ultraviolet B-Mediated Oxidative Damage via the AKT/ERK-NRF2-GSH Pathway In Vitro and In Vivo. Biomol. Ther..

[67] Khan H., Singh A., Singh Y., Sharma D., Dua K., Grewal A.K., Singh T.G. (2025). Pharmacological modulation of PI3K/PTEN/Akt/mTOR/ERK signaling pathways in ischemic injury: A mechanistic perspective. Metab. Brain Dis..

[68] Martono Y., Yanuarsih F., Aminu N., Muninggar J. (2019). Fractionation and Determination of Phenolic and Flavonoid Compound from Moringa oleifera Leaves.

[69] Shraim A.M., Ahmed T.A., Rahman M.M., Hijji Y.M. (2021). Determination of total flavonoid content by aluminum chloride assay: A critical evaluation. LWT.

[70] Re R., Pellegrini N., Proteggente A., Pannala A., Yang M., Rice-Evans C. (1999). Antioxidant activity applying an improved ABTS radical cation decolorization assay. Free Radic. Biol. Med..

[71] Contreras-Guzmán E.S., Strong III F.C. (1982). Determination of tocopherols (vitamin E) by reduction of cupric ion. J. Assoc. Off. Anal. Chem..

[72] Mapoung S., Umsumarng S., Semmarath W., Arjsri P., Srisawad K., Thippraphan P., Yodkeeree S., Dejkriengkraikul P. (2021). Photoprotective effects of a hyperoside-enriched fraction prepared from Houttuynia cordata Thunb. on ultraviolet B-induced skin aging in human fibroblasts through the MAPK signaling pathway. Plants.

