# Supplementary figures and images for: Biochemical Mechanism of Thai Fermented Soybean Extract on UVB-Induced Skin Keratinocyte Damage and Inflammation
Source: Int J Mol Sci. 2025 Apr 5;26(7):3418. doi: 10.3390/ijms26073418 (PMC11989635; doi:10.3390/ijms26073418)

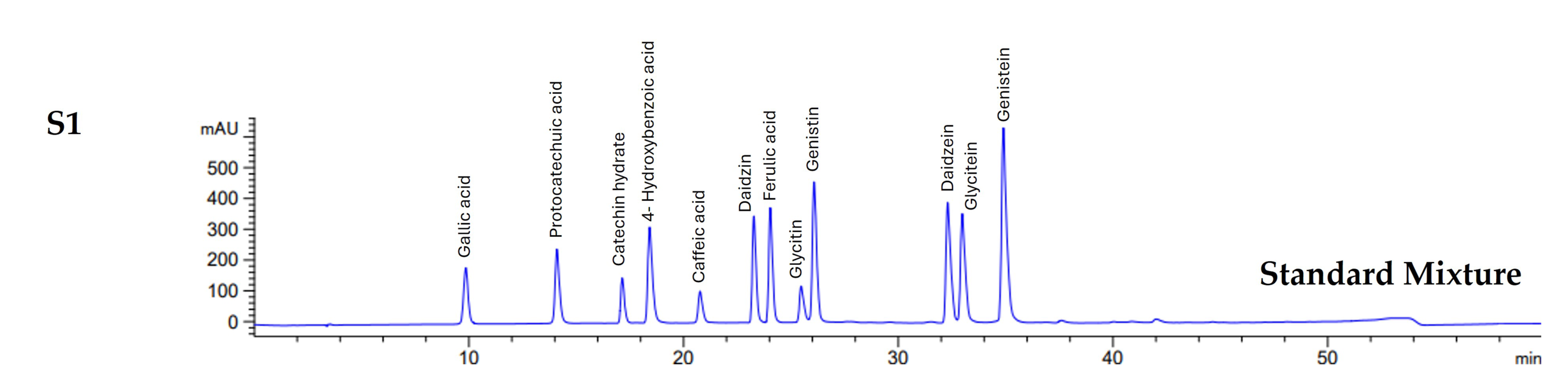

Supplement: Supplementary file 1 [file ijms-26-03418-s001.zip › Figure S1.tif]

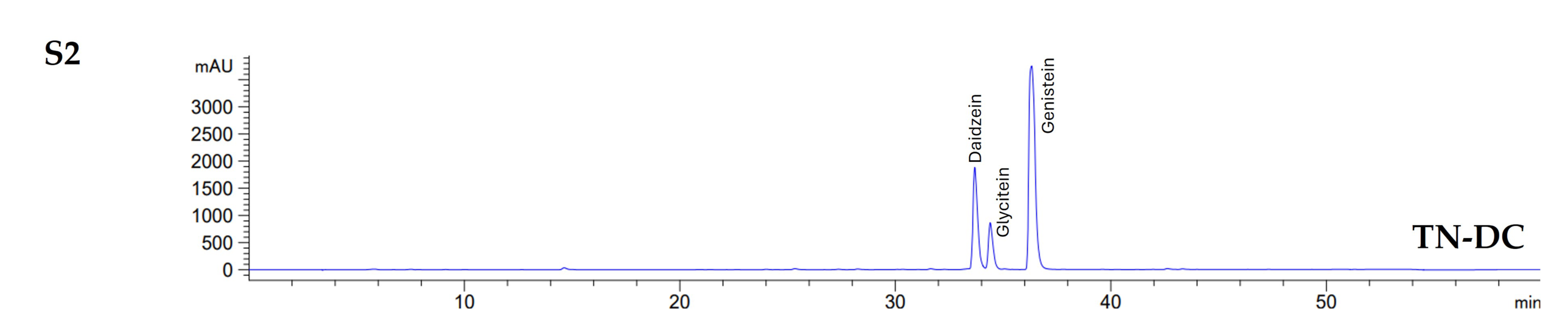

Supplement: Supplementary file 1 [file ijms-26-03418-s001.zip › Figure S2.tif]

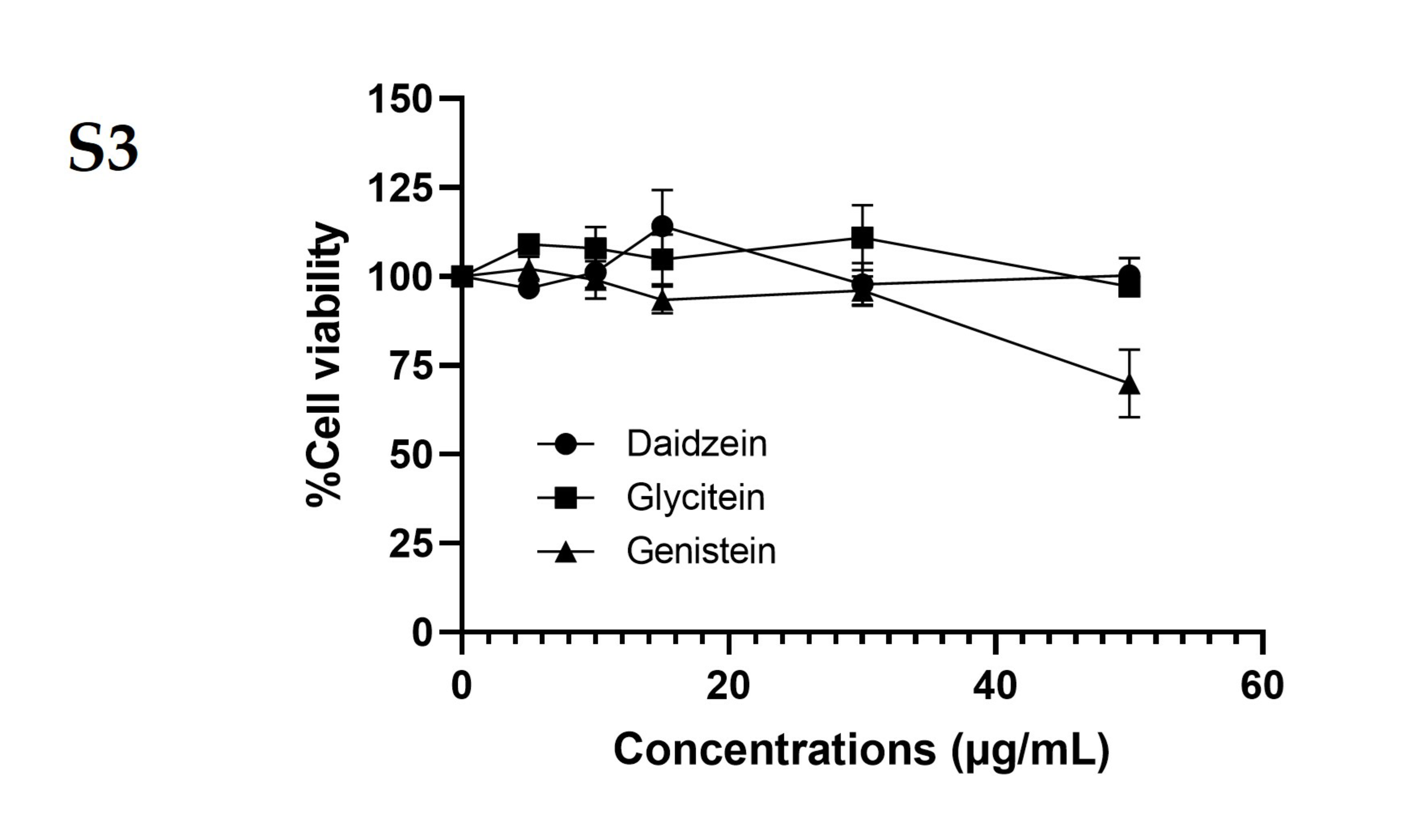

Supplement: Supplementary file 1 [file ijms-26-03418-s001.zip › Figure S3.tif]
